# One-Month Outcomes of Intravitreal Anti-VEGF vs. Dexamethasone Implant in the Treatment of Diabetic Macular Edema in Vitrectomized Eyes

**DOI:** 10.3389/fmed.2022.895220

**Published:** 2022-06-14

**Authors:** Youling Liang, Bin Yan, Manyun Xie, Zhishang Meng, Jiayue Ma, Bosheng Ma, Jing Luo

**Affiliations:** Department of Ophthalmology, The Second Xiangya Hospital, Central South University, Changsha, China

**Keywords:** anti-vascular endothelial growth factor (VEGF), anti-inflammation, diabetic macular edema, vitrectomized eyes, central retinal thickness (CRT)

## Abstract

**Objectives:**

To compare short-term effect of intravitreal ranibizumab with dexamethasone implant for diabetic macular edema (DME) in vitrectomized eyes.

**Methods:**

Single-center, prospective, randomized study of vitrectomized eyes with DME. Study eyes were divided into two groups, receiving ranibizumab (IVV group, *n* = 35 eyes) or dexamethasone implant (IVD group, *n* = 35 eyes) respectively. Patients were evaluated at baseline, Week 1 and Month 1. The main outcome measures included best-corrected visual acuity (BCVA), central retinal thickness (CRT) and intraocular pressure (IOP).

**Results:**

BCVA and CRT were similar in the two groups at baseline. At Week 1, the CRT improvement was significant in two groups (*P* = 0.041 in IVV group, *P* = 0.030 in IVD group), but at Month 1, only IVD group had significant improvement in CRT (*P* < 0.001). And BCVA gains were significant at Week 1 (*P* = 0.029) and Month 1 (*P* = 0.001) in IVD group, whereas IVV group did not show significant BCVA gains (*P* = 0.056 at Week1, *P* = 0.166 at Month 1). The changes of BCVA and CRT were significantly higher in IVD group than IVV group at Month1, but the changes were not significant at Week1.

**Conclusions:**

Comparing to anti-VEGF therapy, DEX implant is more effect in improving BCVA and reducing CRT in vitrectomized eyes at 1 month, which indicated DEX implant is a better strategy.

## Introduction

Diabetic macular edema (DME) is one of the most important causes of visual impairment worldwide and particularly affects working-age individuals (1). Intravitreal injection of therapeutic agents such as anti-vascular endothelial growth factor (VEGF) and steroids was confirmed to be effective in treating DME (2, 3). However, the treatment of DME in vitrectomized eyes is not clearly defined.

Anti-VEGF drugs are currently used as first-line agents for DME, but these drugs are believed to be more rapidly cleared in vitrectomized eyes than in nonvitrectomized eyes (4). The use of intravitreal steroids is the second-line treatment in pseudophakic patients due to the negative effect on lens transparency (5). Additionally, carrier platforms that dissolve within the vitreous over a longer period of time have been developed to prolong the corticosteroid half-life. Ozurdex contains 0.7 mg of dexamethasone (DEX) within its carrying system and can release DEX slowly in the vitreous (6). Ozurdex has been proven to improve both vision and retinal anatomical structure in vitrectomized DME eyes (7). However, intravitreal injection of bevacizumab does not change visual acuity or foveal thickness in vitrectomized eyes (8). In our previous study, we found that inflammatory factors were higher in vitrectomized DME eyes than in nonvitrectomized DME eyes, whereas VEGF was lower in vitrectomized DME eyes than in nonvitrectomized DME eyes (9). These results may indicate that anti-inflammatory therapy is more effective than anti-VEGF therapy in vitrectomized eyes.

This study aimed to find a more effective therapy for vitrectomized DME patients. Depending on some researches that anti-VEGF agent cleaned rapidly in vitrectomized eyes (4), we only compared the short-term effects between Ozurdex and an anti-VEGF agent in vitrectomized eyes for the treatment of DME, including their effects on vision acuity and anatomical structure.

## Methods

This prospective, open-label/subject-masked, randomized, controlled trial was conducted at a single site. Our study was approved by the Ethical Committee of The Second Xiangya Hospital, and all enrolled patients were treated in accordance with the Declaration of Helsinki. All patients provided informed consent before inclusion in the study.

Eligibility for the study was evaluated at a screening visit.

The inclusion criteria were as follows:

- An age of 18-80 years with type 1 or 2 diabetes mellitus (DM).
- A history of pars plana vitrectomy (PPV) at least 1 month before the baseline study visit.
- The presence of clinically significant macular edema (defined as thickening of the retina or hard exudates at ≤ 500 μm from the center of the macula or at least 1 zone of retinal thickening in 1 disc area or larger, any part of which was within 1 disc diameter of the center of the macula).

The exclusion criteria were as follows:

- Intravitreal anti-VEGF injection within the previous 4 weeks, intravitreal triamcinolone injection within the previous 8 weeks, intravitreal DEX (Ozurdex) within the previous 16 weeks.
- The presence of macular ischemia evaluated at baseline by fluorescein angiography (Spectralis/HRA, Heidelberg).
- Active iris neovascularization, aphakia, pseudophakia with anterior chamber intraocular lens, an abnormal vitreoretinal interface that could contribute to secondary macular edema, active or suspected ocular or periocular infections including most viral diseases of the cornea and conjunctiva including active epithelial keratitis due to herpes simplex, herpes zoster, vaccinia, mycobacteria, or fungi.
- A known history of increased intraocular pressure (IOP) because of corticosteroids that would not be adequately controlled with 2 topical glaucoma medications.
- A best-corrected visual acuity (BCVA) ≤ 20/200 on the Snellen scale in the nonstudy eye.
- Uncontrolled systemic disease.
- Any condition that in the opinion of the investigator might compromise the results of the trial or preclude the patient from completing all study visits.
- Pregnancy, nursing, or who might become pregnant.

Eligible subjects were randomly assigned at a 1:1 ratio to one of 2 treatments: (1) intravitreal anti-VEGF agent (IVV) (ranibizumab) or (2) intravitreal DEX delayed delivery system implant (IVD) (Ozurdex). For patients in whom both eyes met the eligibility criteria, the right eye was randomized to a treatment group, and the left eye was assigned to the other group. The study duration was 1 month.

Eyes assigned to the IVV group received intravitreal injection of 0.3 mg of ranibizumab in 0.05 mL at baseline and monthly thereafter if the retreatment criteria were met. Eyes assigned to the IVD group received intravitreal injection of 0.7 mg of the DEX delayed delivery system implant (Ozurdex) at baseline and every 3 months if the retreatment criteria were met. However, we collect data only at Week 1 and Month 1.

Patients received intravitreal injection in the study eye at the baseline visit (Day 1). Other treatments for macular edema in the study eye were prohibited during the study and could be given only if there was concern for patient safety. Patients were seen at outcome assessment study visits on Day 2 and at Week 1 and Month 1.

A complete ophthalmological examination, including BCVA on the Snellen scale (converted into logMAR for statistical comparison), IOP evaluation, slit-lamp biomicroscopy, fundus examination, and spectral-domain optical coherence tomography (OCT; RTVue XR Avanti, Optovue, Inc., Fremont, CA, USA) combined OCT angiography (OCTA), was performed at the initial visit and then repeated at every follow-up visit for all patients (except OCT on Day 2). DME pattern classified according to the presence of intraretinal cysts (IRC) or IRC plus serous retinal detachment (SRD) pattern. The primary efficacy outcomes measure were the change in central retinal thickness (CRT) from baseline to Week 1 and Month 1 measured by OCTA. The key secondary efficacy measure was BCVA.

Statistical analysis was performed using SPSS 24.0 (IBM, Armonk, NY, USA). Categorical variables were described using absolute and relative frequencies, and quantitative variables are reported as the mean ± SD. Mean changes in CRT and BCVA from baseline were analyzed using nonparametric tests, including the Mann–Whitney U test and Fisher's exact *t*-test. *P* < 0.05 were considered to indicate significance.

## Results

A total of 70 eyes in 70 patients (38 male; mean age 51.29 ± 8.99 years) with DME enrolled in the study were assessed. The baseline characteristics of the patients and study eyes are listed in Table 1. Patients had undergone PPV combined with panretinal photocoagulation at 19.15 ± 20.21 weeks on average before study entry, most commonly for vitreous hemorrhage and vitreomacular traction syndrome.

**Table 1:** Baseline characteristics of the two groups.

**Feature**	**IVV Group,** **Mean ±SD**	**IVD Group,** **Mean ±SD**	***P* Value**
Age, years	51.07 ± 10.01	51.67 ± 7.09	0.782
Sex, n			0.321
Male	21	17	
Female	14	18	
Duration of DME, years	10.71 ± 6.28	10.33 ± 5.66	0.806
Time since PPV, weeks	18.22 ± 20.64	20.75 ± 19.80	0.417
Lens status			0.734
Phakic	6	4	
Pseudophakic	29	31	
BCVA, logMAR	0.74 ± 0.33	0.79 ± 0.38	0.758
IOP, mmHg	16.27 ± 2.93	16.67 ± 2.26	0.736
CRT, μm	398.88 ± 105.97	388.00 ± 104.71	0.755
DME patterns			0.597
Intraretinal cysts (IRC)	26	24	
IRC plus serous retinal 10ptdetachment (SRD)	9	11	

In the IVV group, the BCVA was 0.74 ± 0.33 at the baseline visit, 0.68 ± 0.39 at Week 1, and 0.70 ± 0.36 at Month 1. In the IVD group, the BCVA was 0.79 ± 0.38 at the baseline visit, 0.65 ± 0.42 at Week 1, and 0.61 ± 0.34 at Month 1. Compared to the baseline values, the changes in BCVA in the IVV group at Week 1 and Month 1 were not statistically significant (*P* = 0.056 and *P* = 0.146, respectively). In the IVD group, the BCVA changes were statistically significant at Week 1 and Month 1 (*P* = 0.029 and *P* = 0.001, respectively) (Table 2).

**Table 2:** Mean BCVA (logMAR) at baseline and at subsequent time points in the two groups.

**Time point**	***n***.	**IVV Group** **Mean ±SD**	***p***	**IVD Group** **Mean ±SD**	***p***
Baseline	35	0.74 ± 0.33		0.79 ± 0.38	
Week 1	35	0.68 ± 0.39	0.056	0.65 ± 0.42	0.029
Month 1	35	0.70 ± 0.36	0.166	0.61 ± 0.34	0.001

**Figure 1:**
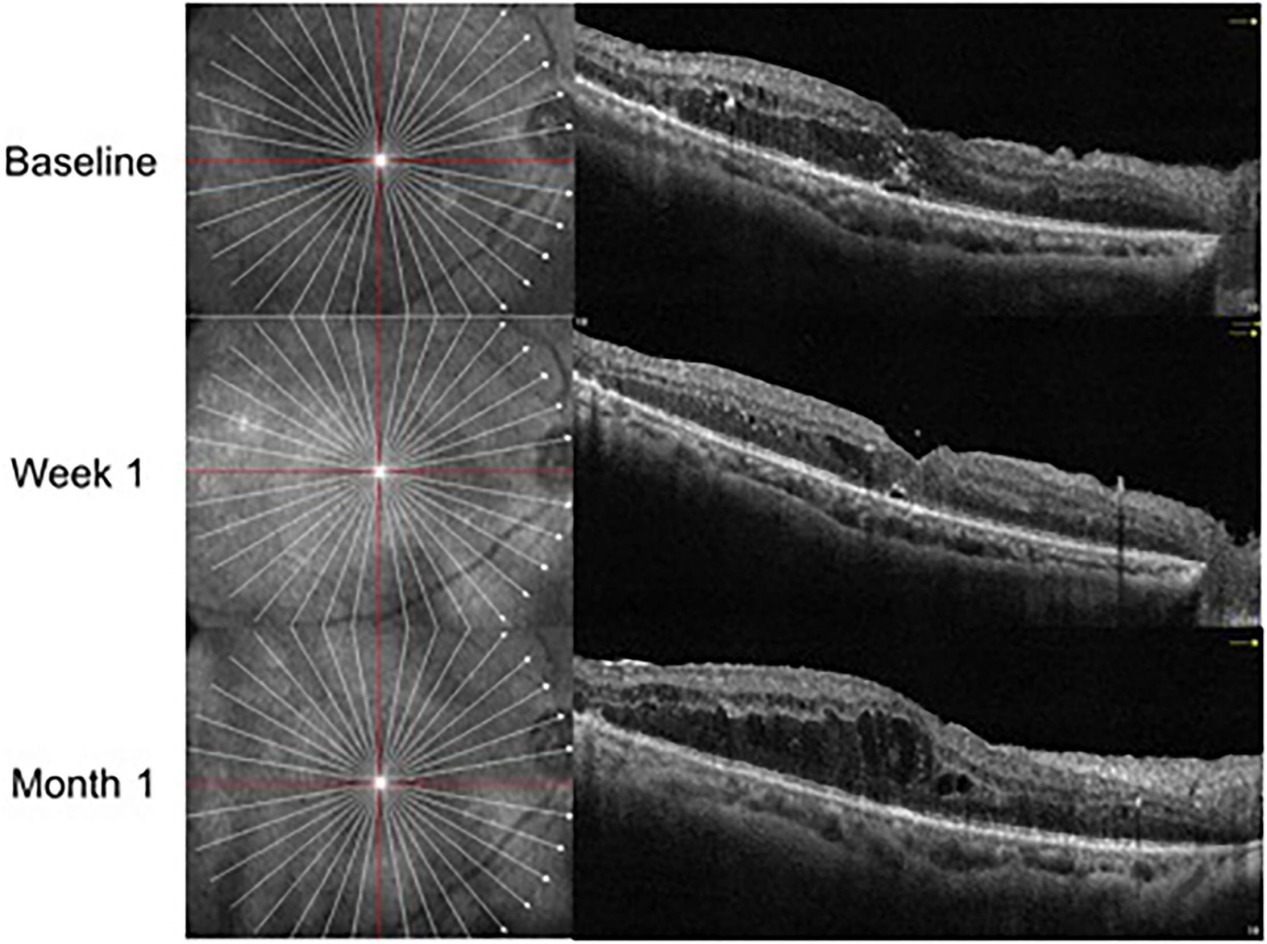
OCT follow-up visit of the same patient with anti-VEGF treatment.

**Figure 2:**
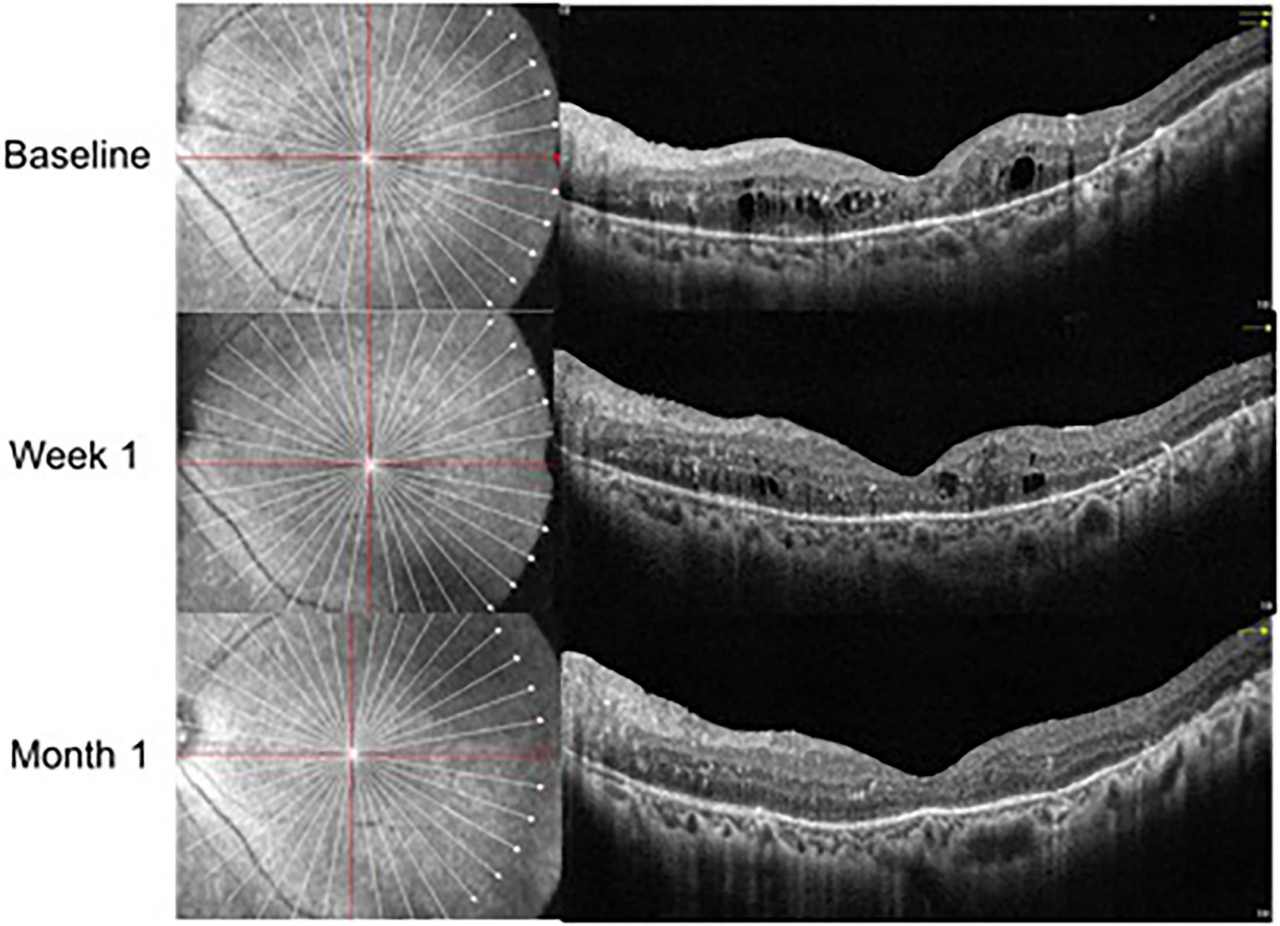
OCT follow-up visit of the same patient with Ozurdex treatment.

**Figure 3:**
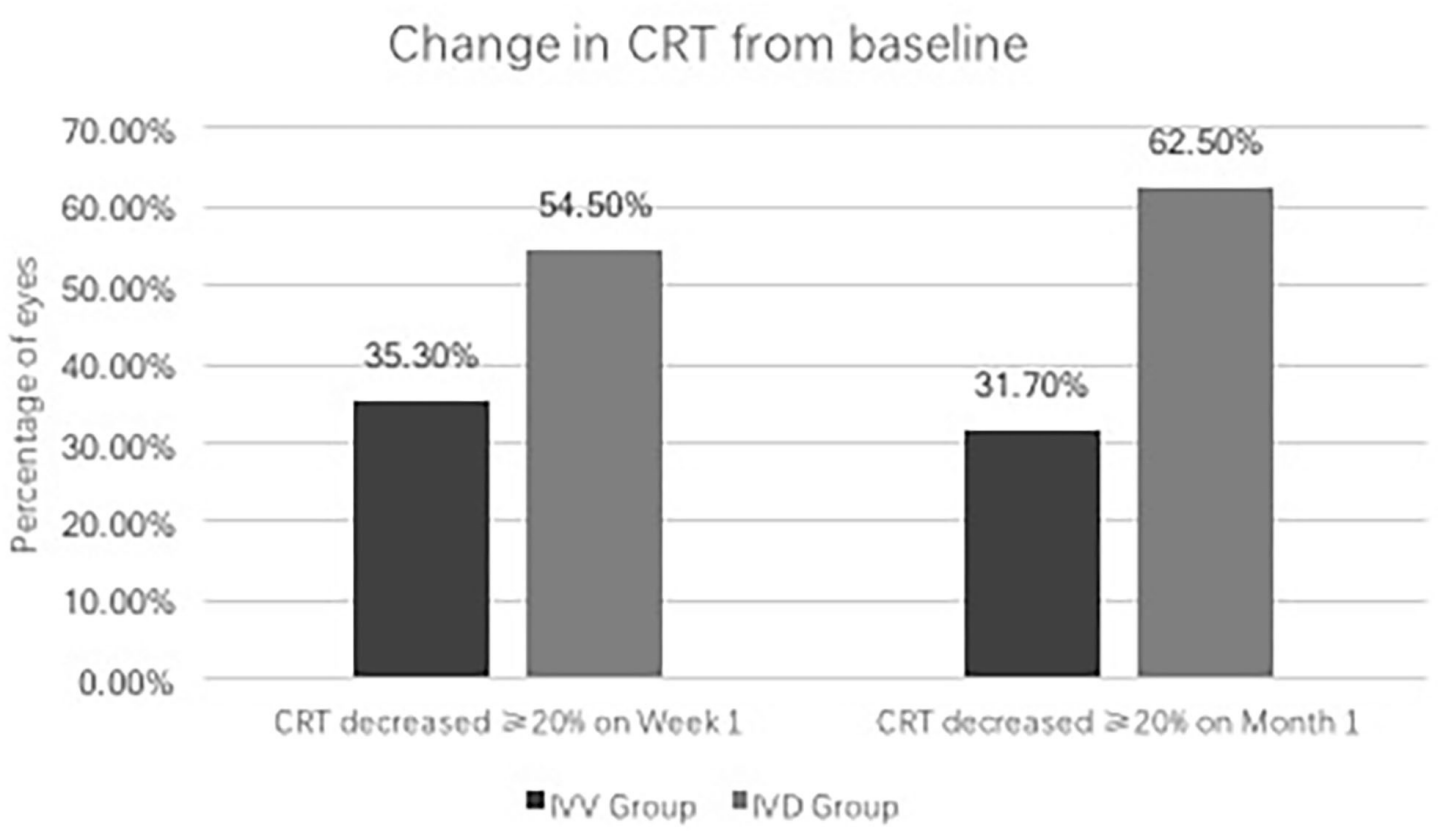
Change in CRT from baseline.

In the IVV group, the mean CRT was 398.88 ± 105.97 μm prior to injection, 333.53 ± 122.43 μm at Week 1, and 382.07 ± 105.49 μm at Month 1 (Figure 1). The changes in CRT were statistically significant at Week 1 but not significant at Month 1 (*P* = 0.041 and *P* = 0.170, respectively). A total of 35.3% of patients in the IVV group had a CRT decrease ≥ 20% at Week 1 and 31.7% at Month 1. 14.6% of patients had CRT worsening ≥ 20% at Month 1, but no patient had CRT worsening ≥ 20% at Week 1. The CRT in the IVD group at the baseline visit was 388.00 ± 104.71 μm, 310.45 ± 69.86 μm at Week 1, and 296.33 ± 48.73 μm at Month 1 (Figure 2). The changes in CRT within Week 1 and Month 1 were statistically significant when compared to the baseline values (*P* = 0.03 and *P* < 0.001, respectively). A total of 54.5% of patients in the IVD group had a decrease in CRT ≥ 20% at Week 1 and 62.5% at Month 1. No patient had CRT worsening ≥ 20% within 1 month (Table 3, Figure 3). The changes in BCVA and CRT between the 2 groups were statistically significant at Month 1 (Table 4).

**Table 3:** Mean CRT (μm) at baseline and subsequent time points in the two groups.

**Time point**	***n***.	**IVV Group** **Mean ±SD**	***p***	**IVD Group** **Mean ±SD**	***p***
Baseline	35	398.88 ± 105.97		388.00 ± 104.71	
Week 1	35	333.53 ± 122.43	0.041	310.45 ± 69.86	0.030
Month 1	35	382.07 ± 105.49	0.170	296.33 ± 48.73	<0.001

**Table 4:** Change in BCVA (logMAR) and CRT (μm) in the two groups.

	**IVV Group**	**IVD Group**	***p***
Change in BCVA at Week 1	−0.13 ± 0.26	−0.16 ± 0.26	0.578
Change in CRT at Week 1	−42.88 ± 79.52	−77.55 ± 55.27	0.186
Change in BCVA at Month 1	−0.04 ± 0.27	−0.18 ± 0.22	0.010
Change in CRT at Month 1	−16.80 ± 125.57	−144.42 ± 90.99	0.001

There was no difference in outcomes between two DME patterns in both two groups. In IVV group, the mean decreasing CRT were −21.14 ± 131.32 μm in IRC DME eyes, and −8.00 ± 116.05 μm in IRC plus SRD DME eyes (*P* = 0.76). In IVD group, the mean decreasing CRT were −95.56 ± 51.34 μm in IRC DME eyes, and −171.00 ± 154.93 μm in IRC plus SRD DME eyes (*P* = 0.078).

High IOP was reported as an adverse event in 8.5% of study eyes in the IVD group. Only 1 patient had an IOP ≥30 mmHg at Month 1, and IOP-lowering medication was initiated. However, none of the eyes required laser or incisional surgery for glaucoma (Table 5). No relevant safety issues were encountered during follow-up in this study.

**Table 5:** Number of eyes with IOP elevation at visits in the IVD Group.

**IOP, mmHg**	**Week 1, *n*(%)**	**Month 1**
>21	1 (2.8%)	1 (2.8%)
>30	0 (0)	1 (2.8%)

## Discussion

This study compared the shot-term response to anti-VEGF agent or Ozurdex in vitrectomized DME patients. Visual and anatomical outcomes were evaluated. A significant improvement in CRT was observed in the IVD group, both at Week 1 and Month 1. However, the improvement in CRT was only observed at Week 1 in the IVV group. Visual acuity was increased in the IVD group at Month 1. However, the improvement in BCVA was not statistically significant in the IVV group at Week 1 nor Month 1.

Blood–retinal barrier breakdown (BRB) leads to vascular leakage into retinal tissue, fluid accumulation, and a decrease in visual acuity, which may be reversible. VEGF has been confirmed to play a vital role in BRB dysfunction, and inflammation can also induce an increase in vascular permeability (10). Therefore, both anti-VEGF and anti-inflammatory treatments are effective in DME patients. However, their treatment effects in vitrectomized DME patients were unconfirmed.

In our study, anti-VEGF treatment decreased CRT within 1 week, but the effect did not persist to Month 1. Visual acuity did not change at any time in the IVV group. This result is partially consistent with a previous study, which showed that neither mean visual acuity nor foveal thickness changed after intravitreal anti-VEGF treatment (8). In this previous study, the results on Week 1 were not shown. We found that anti-VEGF therapy improve anatomical structure at Week 1. However, BCVA had not improved at Week 1. Our results showed that anti-VEGF therapy can improve macular thickness vitrectomized DME patients within a short period.

In contrast, intravitreal injection of DEX implant affected both visual acuity and anatomical outcomes in our study. A previous study demonstrated that treatment with a DEX intravitreal implant led to clinically significant improvements in vitrectomized DME eyes (7). Additionally, this previous study found that after DEX implant injection, improvements in CRT and visual acuity peaked at Week 8 and continued to Week 24. Therefore, the DEX implant is useful in vitrectomized DME eyes and has a persistent effect.

Comparing the two therapies, we found that anti-inflammatory treatment was more effective than anti-VEGF in vitrectomized DME patients at Month 1, but the advantage was not obvious at Week 1. Our previous study demonstrated that the levels of inflammatory factors were higher in vitrectomized DME eyes than in nonvitrectomized DME eyes, and the levels of VEGF were lower in vitrectomized DME eyes than in nonvitrectomized DME eyes (9). These results indicated that inflammation may play an important role in the pathogenesis of DME in vitrectomized eyes. This may be one of the reasons why the DEX implant, a representative anti-inflammatory therapy, is more effective than anti-VEGF agents in vitrectomized DME eyes. High inflammatory status may because of ocular surgery and the reaction to diabetic retinopathy (11, 12). Anti-VEGF agents have been confirmed to have shorter half-lives in vitrectomized eyes than in nonvitrectomized eyes (4). However, the effectiveness of the DEX implant is similar in vitrectomized and nonvitrectomized DME eyes (13). High clearance may be another reason that the DEX implant is more effective than anti-VEGF agents.

According to previous studies, frequently repeated intravitreal injection may be required after PPV surgery because eyes become less viscous after the removal of vitreous and the clearance of intravitreal drugs from the vitreous cavity is accelerated. In our study, the IVV group showed a significant improvement in CRT on Week 1, but on Month 1, the CRT did not change significantly, and the ratio of CRT worsening ≥ 20% was 14.6% at Month 1. This means that anti-VEGF treatment may be effective in vitrectomized DME eyes within a short period, but a greater number of injections are needed if we want to maintain this effect. Therefore, high clearance may partially affect the treatment response to anti-VEGF agents. Another study showed that anti-VEGF injection improved anatomic results, but the rate of improvement was slower in vitrectomized eyes than in nonvitrectomized eyes (14). However, injection of the DEX implant was effective on both Week 1 and Month 1, and a previous study showed that this effect could be sustained for 24 weeks (3). This indicated that inflammation may be a vital pathogenic factor in vitrectomized DME patients, and anti-inflammatory therapy is a more effective method for DME treatment in vitrectomized eyes than anti-VEGF therapy.

Some studies demonstrated that different pattern of DME may affect the CRT changing after intravitreal injection of anti-VEGF agent or Ozurdex (15, 16). Especially the DME associated with SRD may represents a specific inflammatory pattern for which dexamethasone appears to be more effective (17). In our results, after DEX implant injection, changes of CRT in patients with SRD (−171.00 ± 154.93 μm) are greater than eyes without SRD(−95.56 ± 51.34 μm). But the difference was not statistical significant (*P* = 0.078), this may due to the small sample size.

Regarding safety in IVD group, few cases (2/35, 5%) had an IOP elevation that could be managed pharmacologically without any surgical approach. Elevation of IOP is a side-effect for corticosteroid. If treatment with corticosteroid is limited to patients without IOP elevating history due to steroid eye drops, the risk of elevated IOP can be reduced. And patients with previous PPV often receive steroid treatment postoperatively, enabling prediction of whether a steroid response will occur based on findings at that time. That may be the reason of a low incidence of high IOP in our study.

The primary limitation of this study was that we investigated the short-term response to two agents in vitrectomized DME eyes. Depending on the high clearance in vitrectomized eyes, frequent injection of anti-VEGF agents may obtain a persistent therapeutic effect. Because the effective intravitreal interval in uncertain, we only observed 1 month outcomes. Another limitation is the sample size was small, it is because the number of DME patients with PPV history is not that large. Further studies are needed to investigate the effectiveness of multiple injections of the DEX implant for vitrectomized DME patients.

## Conclusion

In conclusion, this study showed that DEX implant is an effective way to treat DME in vitrectomized patients. And anti-VEGF agent can also improve anatomical structure in vitrectomized DME eyes, but the effect lasted less than a month. DEX implant may be a more effective way for DME treatment in vitrecomized eyes.

## Data Availability Statement

The original contributions presented in the study are included in the article/supplementary material, further inquiries can be directed to the corresponding author.

## Ethics Statement

The studies involving human participants were reviewed and approved by Ethical Committee of The Second Xiangya Hospital. The patients/participants provided their written informed consent to participate in this study.

## Author Contributions

YL was responsible for designing the study, conducting the search, screening potentially eligible studies, extracting and analyzing data, interpreting results, and writing the paper. BY, MX, ZM, JM, and BM were responsible for extracting data. JL was responsible for designing the study. All authors contributed to the article and approved the submitted version.

## Funding

This work was supported by the National Natural Science Foundation of China (81570847), Natural Science Foundation of Hunan Province (2020JJ4800).

## Conflict of Interest

The authors declare that the research was conducted in the absence of any commercial or financial relationships that could be construed as a potential conflict of interest.

## Publisher's Note

All claims expressed in this article are solely those of the authors and do not necessarily represent those of their affiliated organizations, or those of the publisher, the editors and the reviewers. Any product that may be evaluated in this article, or claim that may be made by its manufacturer, is not guaranteed or endorsed by the publisher.
